# Effect of Cellgevity® Supplement on Selected Rat Liver Cytochrome P450 Enzyme Activity and Pharmacokinetic Parameters of Carbamazepine

**DOI:** 10.1155/2020/7956493

**Published:** 2020-07-03

**Authors:** Seth Kwabena Amponsah, Benoit Banga N'guessan, Martin Akandawen, Abigail Aning, Sedem Yawa Agboli, Eunice Ampem Danso, Kwabena Frimpong-Manso Opuni, Isaac Julius Asiedu-Gyekye, Regina Appiah-Opong

**Affiliations:** ^1^Department of Pharmacology and Toxicology, School of Pharmacy, College of Health Sciences, University of Ghana, Accra, Ghana; ^2^Department of Clinical Pathology, Noguchi Memorial Institute for Medical Research, College of Health Sciences, University of Ghana, Accra, Ghana; ^3^Department of Pharmaceutical Chemistry, School of Pharmacy, College of Health Sciences, University of Ghana, Accra, Ghana

## Abstract

**Background:**

There is considerable evidence that many patients concurrently administer dietary supplements with conventional drugs, creating a risk for potential drug-supplement interaction. The aim of this study was to determine the effect of Cellgevity® supplement on selected rat liver cytochrome P450 (CYP) enzymes. Also, based on our previous finding, we sought to determine the effect of Cellgevity® on the pharmacokinetics of carbamazepine, a CYP3A4 substrate.

**Methods:**

Male Sprague–Dawley (SD) rats were randomly put into 5 groups and administered either distilled water (negative control), Cellgevity® (3 separate doses), or phenobarbital (positive control), *per os*. Modulation of liver CYP enzyme activity was evaluated after 30 days of treatment, using probe substrates, spectroscopic, and high-performance liquid chromatographic methods. In the pharmacokinetic study, 12 SD rats were put into 2 groups and administered carbamazepine plus normal saline (group 1) or carbamazepine plus Cellgevity® (group 2), *per os*, both over a period of 14 days. Blood samples from rats in the same group were collected after treatment. Serum samples were prepared and pooled together at each specific sampling time point. Levels of carbamazepine were determined using a fluorescence polarization immunoassay.

**Results:**

Activities of rat liver CYP1A1/2, CYP2C9, and CYP2D6 were significantly increased by Cellgevity® after 30-day treatment. Pharmacokinetic parameters for rats administered carbamazepine with Cellgevity® vis-a-vis carbamazepine with normal saline were as follows: *C*_max_; 20 *μ*mol/L vs 11 *μ*mol/L, AUC_0⟶24_; 347 *μ*mol h/L vs 170 *μ*mol h/L, *K*_e_; 0.28 h^−1^ vs 0.41 h^−1^, and *t*_1/2_; 2.3 h vs 1.7 h, respectively.

**Conclusions:**

Cellgevity® increased the activity of rat CYP1A1/2, CYP2C9, and CYP2D6 enzymes and was found to alter the pharmacokinetics of carbamazepine in rats.

## 1. Background

Dietary supplements may be vitamins, minerals, or herbal products that are known to improve the well-being of humans [1]. This clearly denotes the use of these supplements as an addition to dietary requirements that may not be met by daily meals. However, under no circumstance should dietary supplements be used as a replacement for daily meals. The United States Food and Drugs Administration also prohibits the indication of dietary supplements as a treatment for diseases or any indication that connotes dietary supplements as primary therapeutic agents [1].

With an increase in the incidence of noncommunicable diseases (NCDs) such as diabetes, cardiomyopathies, cancers, and epilepsy, which are often associated with oxidative stress, people resort to the use of dietary supplements (known antioxidants) to prevent these diseases [2]. Interestingly, some individuals who use dietary supplements have the notion that these agents may enhance the effects of conventional drugs [3]. This assertion may be false. It is noteworthy, however, that synergy between some dietary supplements and conventional drugs have been reported [4].

Currently available on the market are a number of dietary supplements known to replenish levels of reduced glutathione, a free radical scavenger, in cells of humans. One such supplement is Cellgevity®, which contains a glutathione precursor molecule, riboceine (D-ribose-L-cysteine). Riboceine is known to deliver cysteine into cells and enhance reduced glutathione levels in the body [5, 6]. The other constituents of Cellgevity® are broccoli seed extract, turmeric root extract, resveratrol, grape seed extract, quercetin, curcumin, milk thistle, vitamin C, selenomethionine, cordyceps, black pepper, and aloe extract. Some of these constituents of Cellgevity® are known inducers and/or inhibitors of cytochrome P450 (CYP) enzymes [7, 8]. Cellgevity® is marketed and distributed by Max International, which has branches in 14 countries (United States, Nigeria, Cote d'Ivoire, New Zealand, Singapore, Costa Rica, Columbia, Philippines, El Salvador, Malaysia, Guatemala, Ghana, and Hong Kong). Cellgevity® has gained popularity in these countries due to the fact that patronizers know that the supplement has a high antioxidant potential [9].

Reports suggest that there could be clinically important modulation of CYP enzyme activity by supplements and/or herbal products. This could result in adverse or subtherapeutic effects of concurrently administered conventional drugs. For example, St. John's wort was reported to decrease the serum concentration of theophylline (a bronchodilator) as a result of CYP enzyme induction [10]. This interaction between St John's wort and theophylline could lead to subtherapeutic effect of normal doses of theophylline when there is coadministration. For this reason, patients are often advised not to take theophylline concomitantly with St. John's wort. Most of the individual constituents of Cellgevity® can modulate xenobiotic metabolizing enzyme activity, and several studies have reported these. However, there is paucity of scientific data on the effect of all these constituents (extracts) combined in Cellgevity® on xenobiotic metabolizing enzymes (CYP enzymes). In our earlier study, we reported that Cellgevity® at 4 and 8 mg/kg significantly inhibited rat liver CYP2C9, CYP2B1/2B2, and CYP3A4 after a 7-day treatment period [11]. In animal models, it is relevant that doses of test agents (drugs/food supplements/herbal products) are at equivalent doses of what pertains to humans. Therefore, as a follow-up to our earlier study, we decided to use animal equivalent doses of Cellgevity® after scaling doses used in humans [12] in the current study. We also decided to administer Cellgevity® over a longer period of time, i.e., 30 days.

We have previously reported that Cellgevity® inhibited rat liver CYP3A4 after a 7-day treatment period [11]. We, therefore, sought to elucidate the possible effect of Cellgevity® on carbamazepine, also known to be extensively metabolized by CYP3A4. This was to aid in assessing potential interaction between these two agents. Carbamazepine is one of the most commonly prescribed drugs in the management of epilepsy. Due to the chronic nature of epilepsy, and the fact that patients have to take carbamazepine for a long time (lifetime in most cases), there is the potential for clinically significant interactions between carbamazepine and coadministered agents like dietary supplements, herbal products, and food [13]. Therefore, in a parallel experiment, the effect of Cellgevity® on the pharmacokinetics of carbamazepine (a CYP3A4 substrate) was investigated.

## 2. Methods

### 2.1. Animal Care and Safety

This research was approved by the College of Health Sciences Ethical and Protocol Review Committee (Protocol ID: CHS-Et/M.9–P1.16/2017-2018) of the University of Ghana. All animal procedures used in this study were in accordance with the National Institute of Health Guidelines for the Care and Use of Laboratory Animals [14].

Male Sprague–Dawley (SD) rats, weighing 150–200 g and 6–8 weeks old, were obtained from the Center for Plant Medicine Research, Mampong, Eastern Region, Ghana. The animals were housed in stainless steel cages. Each rat occupied a minimum space of 2 cubic feet (61 cm × 31 cm × 31 cm) with softwood shavings as bedding for their comfort. The SD rats were fed with normal pellet diet (AGRIMAT, Kumasi, Ghana), given water *ad libitum*, and maintained under standard laboratory conditions (temperature ∼25°C, relative humidity 60–70%, and 12 h light-dark cycle). The animals' feeding area and water troughs were cleaned regularly to prevent contamination. Animals were acclimatized under stated conditions for 7 days before the experiment commenced.

### 2.2. Hepatic Enzyme Induction/Inhibition Studies

#### 2.2.1. Animal Grouping and Treatment Administration

In determining the effect of Cellgevity® on CYP enzymes after a 30-day treatment period, male SD rats were put into five groups (6 rats in each group). All treatments were *per os* and for 30 days. Group 1 was administered distilled water, the vehicle used in dissolving Cellgevity® (purchased from Max International, Ghana), and that served as the negative control (N-C) group. Groups 2, 3, and 4 received daily a low dose (L-D) of 38.63 mg/kg Cellgevity®, medium dose (M-D) of 77.25 mg/kg Cellgevity®, and high dose (H-D) of 154.50 mg/kg Cellgevity®, respectively, as reported elsewhere [15]. The doses of Cellgevity® administered to SD rats were animal equivalents of what pertains to humans and calculated as described by Nair AB and Jacob SA [16]. The human dose of Cellgevity® is 12.46 mg/kg *per os*. The SD rats in Group 5 received an oral dose of phenobarbital (Kinapharma, Ghana) 15 mg/kg daily, and that served as the positive control (P-C). After the 30-day treatment period, animals were sacrificed by cervical dislocation. Livers were excised, washed in ice-cold saline solution, weighed, and stored at −80°C until use.

#### 2.2.2. Microsomal Preparation

Livers were thawed and homogenized in potassium phosphate buffer (pH 7.4) using a mortar and pestle on ice. Homogenized samples were first centrifuged at 4,000 rpm for 20 min. The supernatant obtained was recentrifuged (Beckman Avanti J-25, USA) at 25,000 rpm for 2 h. The pellets were obtained, and microsomes were collected and stored at −80^o^C until use.

#### 2.2.3. CYP2C9 (Diclofenac Hydroxylation) and CYP2D6 (Dextromethorphan O-Demethylation) Assays

The assays were performed as previously described [17], with some modification. A volume of 350 *µ*L of 0.1 M potassium phosphate buffer (pH 7.4), 50 *µ*L of 1 mM substrate (diclofenac for CYP2C9 assay and dextromethorphan for CYP2D6, both substrates purchased from Sigma-Aldrich, USA) and 50 *µ*L of 2.5 mg/mL microsome (obtained from rat livers from respective groups) were mixed separately in Eppendorf tubes. The mixtures were preincubated at 37°C for 5 min. A volume of 50 *µ*L of 1 mM nicotinamide adenine dinucleotide phosphate (NADPH) (Sigma-Aldrich, USA) was added, mixed, and incubated at 37°C for 45 min. A 100 *µ*L stopping solution (ZnSO_4_.7H_2_O) was added, and the mixture centrifuged at 4000 rpm for 5 min. The supernatants were aliquoted into High-Performance Liquid Chromatography (HPLC, Shimadzu, Japan) vials.

Samples were analyzed using HPLC. The chromatographic system consisted of a binary solvent delivery system (LC-20AB), a degasser (DGU-20A3), an autosampler (SIL-20ACHT), a column temperature controller (CTO-10AS VP), and a photodiode array detector (SPD - M20 A) for CYP2C9 metabolites and fluorescence detector (RF-10A_XL_) for CYP2D6 metabolites. The following chromatographic conditions were used for the analysis of CYP2C9; column, C18 (Shimadzu, Japan), diameter 5 *μ*m, length *x* width 150 mm × 4.6 mm; flow rate, 1 mL/min; column temperature, 40°C; injection volume, 20 *μ*L; mobile phase, 20 mM potassium phosphate buffer (pH 7.4)/methanol/acetonitrile (60 : 22.5 : 17.5, v/v/v). The same chromatographic conditions were used for the analysis of the CYP2D6, with modification to the mobile phase, where 3 solvents were used (acetonitrile/distilled water/triethylamine; 24 : 75 : 1, v/v/v).

#### 2.2.4. CYP1A1/1A2-Ethoxyresorufin O-Deethylase (EROD), CYP2B1/2B2-Pentoxyresorufin O-Depentylase (PROD), and CYP3A4 - Benzyloxyresorufin O-Debenzylase (BROD) Assays

The assays were performed as previously described [18, 19], with some modification. In brief, microsomes (CYP enzymes) were tested in a total volume of 100 *μ*L. Aliquots of 70 *μ*L potassium phosphate buffer (pH 7.4) were placed into 96-well black plates. This was followed by the addition of 10 *μ*L of 50 *μ*M substrate concentration (resorufin ethyl ether for CYP1A1/2, pentoresorufin for CYP2B1/2 and resorufin benzyl ether for CYP3A4; all substrates purchased from Sigma-Aldrich, USA). The final substrate concentration in 100 *μ*L total reaction volume was 5 *μ*M with 0.25% (v/v) dimethyl sulfoxide (DMSO). It is noteworthy that CYP activities were not expected to be affected at the DMSO concentration used in this experiment [20]. Aliquots of 10 *μ*L enzyme (microsome from each rat liver from respective groups) corresponding to 1 mg/mL protein concentration and the vehicle were added in triplicate. The mixtures were preincubated at 37°C for 5 min. A volume of 10 *μ*L of NADPH was then added to each well, and the setup was incubated for 10 min for CYP1A1/2, 20 min for CYP2B1/2, and 30 min for CYP3A4 assays, respectively. Aliquots of 40 *μ*L of stopping solution (20% 0.5 M Tris: 80% acetonitrile) were added to each well and shaken gently. Fluorescence of wells was read at wavelengths of 530 nm excitation and 586 nm emission. Triplicate experiments were performed. The average absorbance of the blank was subtracted from the average absorbance of each sample.

### 2.3. Fourteen-Day Treatment of Cellgevity® on the Pharmacokinetics of Carbamazepine in Rats

#### 2.3.1. Animal Grouping and Treatment Administration

Twelve male SD rats were obtained for this aspect of the study. The animals were put into 2 groups of 6 (Group 1 and Group 2). All treatments were *per os* and for 14 days. Group 1 was administered carbamazepine plus saline and Group 2, Cellgevity® plus carbamazepine. A dose of 77.25 mg/kg/day of Cellgevity® plus 80 mg/kg of carbamazepine, both equivalent doses per serving/administration in humans scaled to SD rats [14], were administered to rats in Group 2. Rats in Group 1 received 80 mg/kg/day of carbamazepine plus normal saline (same volume as calculated per rat for the Cellgevity® dose).

#### 2.3.2. Blood Sample Collection

After administration of carbamazepine with or without Cellgevity® every 24 h for 14 consecutive days to Groups 1 and 2, tail vein blood samples were taken following the dose administered on the 14^th^ day. Samples were drawn after 0.5, 1, 4, 12, and 24 h. Blood was collected into microtainer gel tubes and centrifuged at 2000 rpm for 5 min to separate serum, and this was stored at −20°C until analysis.

#### 2.3.3. Assay for Carbamazepine in Serum

Due to low sample volumes from tail veins of SD rats, serum samples from rats in the same group (6 animals) at each time point were pooled together. Such that, for instance, serum samples of Group 1 SD rats at time 0.5, 1, 4, 12, or 24 h, were pooled together to obtain a single sample. Usually, challenges with low sample volume can be circumvented by the approach of sample-pooling [21]. Analysis of carbamazepine in serum was by fluorescence polarization immunoassay (FPIA) (Cobas Integra® 400 Plus, Roche, Philippines). The lower limit of quantification of serum carbamazepine concentration was 0.8 *µ*mol/L and the coefficient of variation was <5% over the entire calibration range.

### 2.4. Statistical Analysis

CYP activity of treatment groups was expressed as a percentage relative to the negative control group. All values were expressed as mean ± standard deviation. Differences between groups were tested for significance using a One-Way Analysis of Variance (ANOVA). This was followed by post hoc analysis using Bonferroni's Multiple Comparison Tests. *p*-values < 0.05 were considered statistically significant.

Noncompartmental pharmacokinetic analysis was used to estimate the various pharmacokinetic parameters of carbamazepine. The maximum serum drug concentration (*C*_max_) and its corresponding time (*T*_max_) were determined by visual inspection of the concentration-time curve. The linear trapezoidal rule was applied in extrapolating area under the concentration-time curves (AUCs) for the two groups. The elimination rate constant (*K*_e_) for both groups was extrapolations (apparent slope) from the last sample time point, i.e., 24 hours. *K*_e_ for both groups was used to calculate corresponding elimination half-lives (*t*_1/2_). Pharmacokinetic analysis was conducted using GraphPad Prism 7.0.

## 3. Results

### 3.1. Hepatic Enzyme Induction/Inhibition Studies

All CYPs enzyme activities in the treatment groups were estimated relative to the negative control (N-C) group.

#### 3.1.1. CYP2C9 Activity in SD Rats after 30-Day Treatment

**Figure 1:**
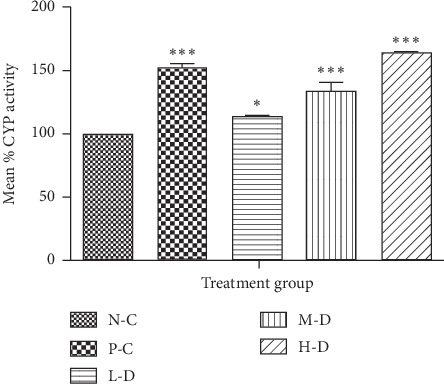
Rat liver CYP2C9 activity for various treatment groups after 30-day administration: N-C = Negative control; P-C = Positive control (Phenobarbital); L-D = Low dose (38.63 mg/kg) of Cellgevity®; M-D = Medium dose (77.25 mg/kg) of Cellgevity®; H-D = High dose (154.50 mg/kg) of Cellgevity®. Data represent mean ± standard deviations. ^*∗*^ and ^*∗∗∗*^ represent values significantly different from the negative control as indicated by *p* < 0.05 and *p* < 0.01, respectively.

CYP2C9 enzyme activity was higher in the phenobarbital- and Cellgevity®-treated groups in comparison with the N-C group. The phenobarbital- and Cellgevity®-treated groups were significantly different from the N-C group. The increase in rat CYP2C9 enzyme activity by Cellgevity® was dose-dependent. Presentation of the effect of Cellgevity® on rat CYP2C9 enzyme activity in various groups is shown in Figure 1.

#### 3.1.2. CYP2D6 Activity in SD Rats after 30-Day Treatment

**Figure 2:**
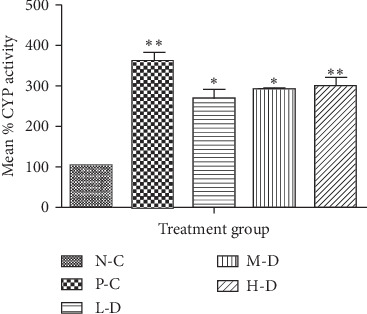
Rat liver CYP2D6 activity for various treatment groups after 30-day administration: N-C = Negative control; P-C = Positive control (Phenobarbital); L-D = Low dose (38.63 mg/kg) of Cellgevity®; M-D = Medium dose (77.25 mg/kg) of Cellgevity®; H-D = High dose (154.50 mg/kg) of Cellgevity®. Data represent mean ± standard deviations. ^*∗*^ and ^*∗∗*^ represent values significantly different from the negative control as indicated by *p* < 0.05 and *p* < 0.01, respectively.

CYP2D6 enzyme activity was about 2.5-fold higher in the phenobarbital-treated and about 2-fold higher in the Cellgevity®-treated groups in comparison with the N-C group. The positive control (P-C) and H-D Cellgevity®-treated groups differed significantly (*p* < 0.01) from the N-C group. The L-D and M-D groups also differed significantly (*p* < 0.05) in comparison to the N-C group. The increase in rat CYP2D6 enzyme activity by Cellgevity® was not dose-dependent. CYP2D6 enzyme activity of SD rats in various groups after the 30-day treatment period is shown in Figure 2.

#### 3.1.3. CYP1A1/2 Activity in SD Rats after 30-Day Treatment

**Figure 3:**
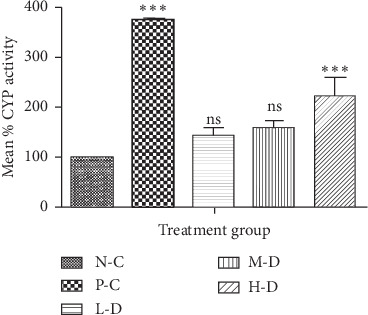
Rat liver CYP1A1/2 activity for various treatment groups after 30-day administration: N-C = Negative control; P-C = Positive control (Phenobarbital); L-D = Low dose (38.63 mg/kg) of Cellgevity®; M-D = Medium dose (77.25 mg/kg) of Cellgevity®; H-D = High dose (154.50 mg/kg) of Cellgevity®. Data represent mean ± standard deviations. ^*∗∗∗*^ represents values significantly different from the negative control as indicated by *p* < 0.001, and ns means not significantly different.

CYP1A1/2 enzyme activity was higher in the phenobarbital- and Cellgevity®-treated groups in comparison with the N-C group. The Cellgevity®-treated L-D and M-D groups showed elevated CYP activity compared to the N-C group, but these differences were not statistically significant. However, there was a significant difference between the H-D Cellgevity®-treated group and the N-C group. There was somewhat a dose-dependent increase in rat CYP1A1/2 enzyme activity in the Cellgevity®-treated group. CYP1A1/2 enzyme activity of SD rats in various groups after the 30-day treatment is shown in Figure 3.

#### 3.1.4. CYP2B1/2 and CYP3A4 Activity in SD Rats after 30-Day Treatment

**Figure 4:**
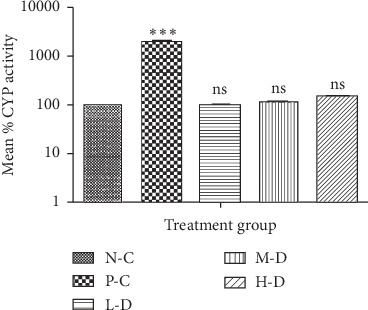
Rat liver CYP2B1/2 activity for various treatment groups after 30-day administration: N-C = Negative control; P-C = Positive control (Phenobarbital); L-D = Low dose (38.63 mg/kg) of Cellgevity®; M-D = Medium dose (77.25 mg/kg) of Cellgevity®; H-D = High dose (154.50 mg/kg) of Cellgevity®. Data represent mean ± standard deviations. ^*∗∗∗*^ represents values significantly different from the negative control group as indicated by *p* < 0.001, and ns means not significantly different.

**Figure 5:**
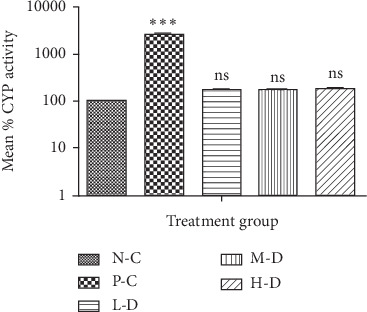
Rat liver CYP3A4 activity for various treatment groups after 30-day administration: N-C = Negative control; P-C = Positive control (Phenobarbital); L-D = Low dose (38.63 mg/kg) of Cellgevity®; M-D = Medium dose (77.25 mg/kg) of Cellgevity®; H-D = High dose (154.50 mg/kg) of Cellgevity®. Data represent mean ± standard deviations. ^*∗∗∗*^ represents values significantly different as indicated by *p* < 0.001, and ns means not significantly different.

There was no statistically significant difference between CYP2B1/2 and CYP3A4 enzyme activities in Cellgevity®-treated groups when compared with the N-C group. CYP2B1/2 and CYP3A4 enzyme activities of SD rats in various groups after 30-day treatment are shown in Figures 4 and 5.

#### 3.1.5. Overall Effect of Cellgevity® on Rat CYP Enzyme Activity

After 30 days, when the Cellgevity®-treated groups were compared to the N-C group, the activities of CYP3A4 and CYP2B1/2 did not differ significantly. However, CYP1A1/2, CYP2C9, and CYP2D6 activities in SD rats treated with Cellgevity® were significantly increased compared to the N-C group. Additionally, the increase in CYP2C9 activity was dose-dependent. The overall effect of Cellgevity® on selected rat CYP enzymes is shown in Table 1.

### 3.2. Fourteen-Day Treatment of Cellgevity® on the Pharmacokinetics of Carbamazepine in Rats

**Figure 6:**
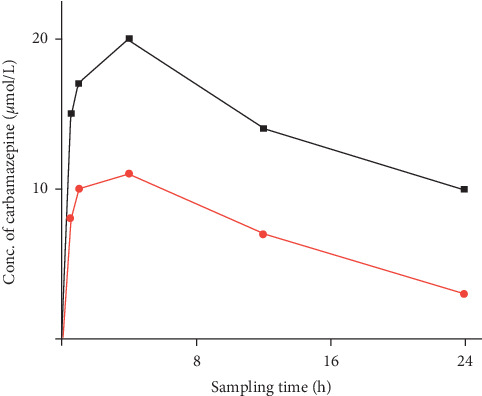
Concentration-time curves of carbamazepine with Cellgevity® (grey solid line; filled squares) and carbamazepine with normal saline (red solid line; filled circles). Serum samples from rats in the same group (*n* = 6) were pooled together at each sampling time point.

Carbamazepine concentration-time curves of rats administered carbamazepine with Cellgevity® and carbamazepine with normal saline are shown in Figure 6. From the concentration-time curves, rats administered carbamazepine with Cellgevity® had a higher peak concentration at 4 h compared to the rats administered carbamazepine with normal saline.

The total carbamazepine exposure (AUC_0⟶24_) and peak carbamazepine concentration for rats administered carbamazepine with normal saline were 170 *µ*mol h/L and 11 *µ*mol/L, respectively, as against 347 *µ*mol h/L and 20 *µ*mol/L, respectively, for rats administered carbamazepine with Cellgevity®. Elimination rate constants were 0.28 h^−1^ for SD rats administered carbamazepine with Cellgevity® and 0.41 h^−1^ for SD rats administered carbamazepine with normal saline. Pharmacokinetic parameters obtained from the concentration-time curves for the two groups are shown in Table 2.

## 4. Discussion

We earlier reported the potential of Cellgevity® to modulate CYP enzymes in rats [11]. In that study, 4 mg/kg and 8 mg/kg of Cellgevity® were admistered to SD rats over a period of 7 days. Cellgevity® inhibited rat liver CYP3A4, CYP2C9, and CYP1A2, after the 7-day treatment period [11]. In the current study, an equivalent dose of Cellgevity® per serving in humans (12.46 mg/kg) was scaled to SD rats [16], and three doses were used over a period of 30 days. Reports suggest that xenobiotics can modulate CYP enzymes depending on dose and treatment duration [12]. This study, therefore, sought to investigate the effect of Cellgevity® on rat liver CYP enzymes using 38.63 mg/kg, 77.25 mg/kg, and 154.50 mg/kg of Cellgevity® calculated after scaling from humans and administering Cellgevity® for 30 days.

In the present study (after the 30-day treatment period), Cellgevity® significantly increased the activity of rat CYP1A1/2, CYP2C9, and CYP2D6. These results differ from what we earlier reported [11], where Cellgevity® significantly inhibited rat CYP3A4, CYP2B1/2B2, and CYP2C9 after a 7-day treatment period. Horn et al. [12] showed that CYP activity can be modulated by both dose and treatment duration. Pichard-Garcia et al. [22] reported that higher concentrations of eletriptan induced CYP3A in culture medium; however, lower concentrations did not cause CYP3A induction. Organisms after exposure to xenobiotics or foreign chemicals often develop adaptive mechanisms where they increase metabolism in an attempt to get rid of the insulting agent. This adaptive mechanism may have accounted for the increased activity of rat CYP1A1/2, CYP2C9, and CYP2D6 observed when relatively higher doses of Cellgevity® (38.63 mg/kg, 77.25 mg/kg, and 154.50 mg/kg) were used in the current study as compared to our earlier study (4 mg/kg and 8 mg/kg of Cellgevity®).

Indeed, it may not be entirely prudent to extrapolate animal studies to humans, but these data give credence to the fact that dietary supplements could modulate CYP enzymes in humans. If this increase in rat CYP1A1/2, CYP2C9, and CYP2D6 (dose-dependent in the case of CYP2C9) activity is of clinical relevance, then emphasis should be made on maximum daily doses of Cellgevity® in humans.

There are reports of potential interaction between dietary supplements/herbal products and conventional drugs. The commonest of these interactions appear to occur at the level of drug metabolism, especially with liver microsomal enzymes. We earlier reported that Cellgevity® significantly inhibited rat liver CYP3A4, CYP2C9, and CYP2B1/2B2 after a 7-day treatment period [11]. On the premise that CYP3A4 was inhibited by Cellgevity® in our earlier research, the current study also sought to determine the effect of Cellgevity® on the pharmacokinetics of carbamazepine (a CYP3A4 substrate) after a 14-day treatment period in SD rats. The peak concentration for rats administered carbamazepine with Cellgevity® was about 2-fold greater than rats administered carbamazepine with saline. Total drug exposure at the last sample time point (AUC_0⟶24_) was also about 2-fold greater in rats administered carbamazepine with Cellgevity® compared to carbamazepine with saline. This meant that there was a relatively slower elimination of carbamazepine in rats administered carbamazepine with Cellgevity®, possibly via inhibition of rat CYP 3A activity by Cellgevity®. This ultimately led to a longer half-life (2.3 h) among rats administered carbamazepine with Cellgevity®. Although the current study using an animal model showed some level of interaction between Cellgevity® and carbamazepine, a limitation was the inadequate sample volumes at each time point, which led to the pooling of serum. Therefore, mean pharmacokinetic parameters within each group could not be obtained for statistical comparison. Notwithstanding, a comparison of pharmacokinetic parameters of traditional versus pooled samples has found no statistically significant difference between the two sets of parameter estimates [21]. It can, therefore, be inferred from the current study that Cellgevity® had some level of interaction with carbamazepine, possibly through inhibition of CYP3A, the enzyme known to metabolize carbamazepine.

In a related study, anecdotal reports suggested that epileptic patients were taking diosmin, a widely used flavonoid in the treatment of varicose veins and haemorrhoids, along with carbamazepine. This led to a study to ascertain possible interaction between these two agents in an animal model [23]. *C*_max_, AUC, and *t*_1/2_ of carbamazepine were significantly elevated in diomin-treated rats compared to control rats [23]. This, therefore, corroborates findings from the current study that there is the potential for herbal medicines, dietary supplements, and food to interact with conventional drugs *in vivo* [24], and that studies of this nature ought to be conducted to identify potential herb-drug interactions.

## 5. Conclusion

In the current study, Cellgevity® caused an appreciable increase in the activities of rat liver CYP1A1/2, CYP2C9, and CYP2D6 enzymes after a 30-day treatment period. Additionally, Cellgevity® altered the pharmacokinetics (elimination rate and half-life) of carbamazepine in Sprague–Dawley rats after a 14-day treatment. Although this study was conducted in an animal model, this finding is noteworthy, as this may serve as a basis for future studies, i.e., assessing the effect of Cellgevity® on protein content and/or mRNA of distinct CYP proteins in rat livers and direct effect of Cellgevity® on recombinant human CYP enzymes.

## Figures and Tables

**Table 1:** Summary of the effect of Cellgevity® on selected rat CYP enzyme activity.

CYP isoform	Assay	Effect of Cellgevity® on CYP activity
CYP3A4	BROD	No significant increase in enzyme activity
CYP2B1/2	PROD	No significant increase in enzyme activity
CYP1A1/2	MROD	Significant increase in enzyme activity (H-D: *p* < 0.001)
CYP2C9	Diclofenac hydroxylation	Significant increase in enzyme activity (L-D: *p* < 0.05; M-D and H-D: *p* < 0.001)
CYP2D6	Dextromethorphan O-Demethylation	Significant increase in enzyme activity (L-D and M-D: *p* < 0.05; H-D: *p* < 0.01)

L-D = Low dose (38.63 mg/kg) of Cellgevity®; M-D = Medium dose (77.25 mg/kg) of Cellgevity®; H-D = High dose (154.50 mg/kg) of Cellgevity®.

**Table 2:** Pharmacokinetic parameters of carbamazepine for the two groups of rats administered carbamazepine plus Cellgevity®, or carbamazepine plus normal saline over a period of 14 days.

Parameters (unit)	Carbamazepine + normal saline	Carbamazepine + cellgevity®
*C*_max_ (*µ*mol/L)	11	20
*T*_max_ (h)	4	4
*k*_e_ (h^−1^)	0.41	0.28
*t*_1/2_ (h)	1.7	2.3
AUC_0_ ⟶ _24_ (*µ*mol h/L)	170	347
AUC_0_ ⟶ _∞_ (*µ*mol h/L)	178	375

## Data Availability

Data used to support the findings of this study are available from the corresponding author upon request.

## Abbreviations

ANOVA: One-way analysis of variance
AUC: Area under the concentration-time curve
*C*_max_: Maximum serum drug concentration
CYP: Cytochrome P450 enzymes
DMSO: Dimethyl sulfoxide
FPIA: Fluorescence polarization immunoassay
H-D: High dose
HPLC: High-performance liquid chromatography
*K*_e_: Elimination rate constant
L-D: Low dose
M-D: Medium dose
NADPH: Nicotinamide adenine dinucleotide phosphate
N-C: Negative control
NCD: Noncommunicable disease
P-C: Positive control
rpm: Revolutions per minute
SD: Sprague–Dawley
*t*_1/2_: Elimination half-life
*T*_max_: Time to reach maximum serum drug concentration.
